# Prognostic significance of the bcl-2 apoptotic family of proteins in primary and recurrent cervical cancer.

**DOI:** 10.1038/bjc.1998.466

**Published:** 1998-07

**Authors:** R. A. Crawford, C. Caldwell, R. K. Iles, D. Lowe, J. H. Shepherd, T. Chard

**Affiliations:** St. Bartholomew's Hospital, London, UK.

## Abstract

bcl-2 is one of a family of genes that control the apoptotic threshold of a cell. bcl-2 protein and its anti-apoptotic homologue, mcl-1, with the pro-apoptotic protein, bax, are thought to function by forming homo- and heterotypic dimers that then control the progression to apoptosis. p53 is also involved as a down-regulator of bcl-2 and a promoter of bax. To determine the effect of these apoptotic mechanisms, we used immunohistochemistry to determine the prognostic significance of the expression of bcl-2, mcl-1, bax and p53 in primary and recurrent cervical cancer. Tissues from 46 patients with primary cervical cancer and 28 women with recurrent carcinoma were stained for bcl-2, mcl-1, bax and p53. Kaplan-Meier survival analysis was performed using the log-rank test for differences between groups. In the primary disease group, positive staining for bcl-2 was associated with a better 5-year survival (bcl-2 +ve, 84% vs bcl-2 -ve, 53%, P = 0.03). Positive staining for p53 was associated with a survival disadvantage (p53 +ve, 4-year survival 38% vs p53 -ve, 4-year survival 78%, P = 0.02). mcl-1 and bax staining were not useful as prognostic indicators in primary disease. No marker was prognostic in recurrent disease. Positive bcl-2 staining defines a group of patients with primary disease with a good prognosis. p53, an activator of the bax promoter, identifies a group with a worse outcome. In recurrent disease, none of the markers reflected prognosis.


British Journal of Cancer (1998) 78(2), 210–214
© 1998 Cancer Research Campaign

### Prognostic significance of the bcl-2 apoptotic family of proteins in primary and recurrent cervical cancer

**RAF Crawford, C Caldwell, RK Iles, D Lowe, JH Shepherd and T Chard**

St. Bartholomew's Hospital, London, UK

*bcl-2* is one of a family of genes that control the apoptotic threshold of a cell. bcl-2 protein and its anti-apoptotic homologue, mcl-1, with the pro-apoptotic protein, bax, are thought to function by forming homo- and heterotypic dimers that then control the progression to apoptosis. p53 is also involved as a down-regulator of bcl-2 and a promoter of bax. To determine the effect of these apoptotic mechanisms, we used immunohistochemistry to determine the prognostic significance of the expression of bcl-2, mcl-1, bax and p53 in primary and recurrent cervical cancer. Tissues from 46 patients with primary cervical cancer and 28 women with recurrent carcinoma were stained for bcl-2, mcl-1, bax and p53. Kaplan–Meier survival analysis was performed using the log-rank test for differences between groups. In the primary disease group, positive staining for bcl-2 was associated with a better 5-year survival (bcl-2 +ve, 84% vs bcl-2 –ve, 53%, $P = 0.03$). Positive staining for p53 was associated with a survival disadvantage (p53 +ve, 4-year survival 38% vs p53 –ve, 4-year survival 78%, $P = 0.02$). mcl-1 and bax staining were not useful as prognostic indicators in primary disease. No marker was prognostic in recurrent disease. Positive bcl-2 staining defines a group of patients with primary disease with a good prognosis. p53, an activator of the bax promoter, identifies a group with a worse outcome. In recurrent disease, none of the markers reflected prognosis.

**Keywords:** bcl-2; mcl-1; bax; p53; primary and recurrent cervical cancer

The process of carcinogenesis may be associated with increased stimulation of cell growth, loss of growth suppression, alterations in immune surveillance and changes in apoptosis. The *bcl-2* gene, located on chromosome 18, encodes a 25-kDa protein that is localized to the mitochondria, smooth endoplasmic reticulum and perinuclear membrane (Hockenberry et al, 1990). bcl-2 has been found to prevent apoptosis (programmed cell death) instead of promoting cell proliferation and thus represents a new class of oncogenes (Yang and Korsmeyer, 1996).

All mammalian cells express several cell death proteases such as the bcl-2 family of cell death regulators, even when they are not undergoing apoptosis (Vaux and Strasser, 1996). Overexpression of bcl-2 specifically prevents cells from initiating apoptosis in response to a number of stimuli, whereas it has little or no ability to promote cell cycle progression or cell proliferation. The ability of bcl-2 to inhibit apoptosis is dependent on expression of *bcl-2* and on the formation of hetero- and homodimers between members of the bcl-2 family (Yang et al, 1995; Kernohan and Cox, 1996). In vivo, bcl-2 associates with a 21-kDa protein known as bax (*b*cl-2-*a*ssociated protein *X*), with which it shares extensive amino acid homology. Oltvai et al (1993) demonstrated that bcl-2 forms heterodimers with bax. The bax–bax homodimer favours cell death, whereas the bax–bcl-2 heterodimer promotes cell viability. The *mcl-1* gene (*m*yeloid *c*ell *l*eukaemia-1) encodes a 37-kDa protein that has significant homology with bcl-2. Like bcl-2, it can partially abrogate apoptosis by blocking bax, although weaker in action. mcl-1 has been localized to the upper layer of the stratified squamous epithelium (Krajewski et al, 1994, 1995). mcl-1 and bcl-2 are expressed at different stages of differentiation in many normal tissues.

*p53*, located on chromosome 17, encodes a 53-kDa protein that is involved in cell growth regulation. In addition, p53 can induce apoptosis in some cells and can down-regulate bcl-2 (Miyashita et al, 1994). Induction of *p53* alone is insufficient to trigger apoptosis but it can increase the sensitivity of the tumour cells to apoptosis induced by DNA damage. DNA damage leads to increased *p53* expression. In addition, *p53* can activate the bax promoter, thus shifting the bcl-2–bax ratio to a state of bax excess, favouring apoptosis.

Expression of bcl-2 is associated with better survival in patients with solid tumours. There appears to be an inverse relationship between p53 and bcl-2 expression in breast cancer (Haldar et al, 1994), ovarian cancer (Henriksen et al, 1995) and lymphoma (Nguyen et al, 1996). This suggests an interaction between these two factors in the regulation of programmed cell death. Several studies have examined the role of bcl-2 either alone (Tjalma et al, 1997) or in conjunction with bax (Uehara et al, 1995) in cervical cancer. Tjalma et al (1997) demonstrated a survival advantage for bcl-2-positive patients, whereas Uehara et al (1995) showed no prognostic significance of either bcl-2 or bax staining.

The objective of this study was to examine the prognostic significance of immunohistochemical detection of the bcl-2 family of apoptotic proteins (bcl-2, mcl-1 and bax) and p53 in primary and recurrent cervical cancer.

*Received 24 July 1997*
*Revised 5 January 1998*
*Accepted 16 January 1998*

*Correspondence to:* RAF Crawford, Department of Gynaecology, St. Bartholomew's Hospital, West Smithfield, London EC1A 7BE, UK

### MATERIALS AND METHODS

The patients were a consecutive group of women attending the gynaecological oncology units at St. Bartholomew's and The Royal Marsden Hospitals, London, from January 1990 until September 1992. Local Ethics Committees' permission was given

**Table 1** Conditions for individual antibodies

| Protein | Antibody | Dilution and exposure | Antigen pretreatment | Control | Staining |
|---|---|---|---|---|---|
| bcl-2[a] | Dako 124 mouse MC | 1/50 40 min | Pressure cooking | Tonsil; internal | Cytoplasmic |
| mcl-1 | Pharmingen rabbit PC 13656E | 1/2000 overnight | Pressure cooking | Tonsil; internal | Cytoplasmic |
| bax | Santa Cruz N20 rabbit PC | 1/500 overnight | None | Tonsil; internal | Cytoplasmic |
| p53* | Dako DO-7 mouse MC | 1/100 overnight | None | Breast | Nuclear |

[a]Antibodies in daily routine use in the diagnostic laboratory. MC, monoclonal; PC, polyclonal.

**Table 2** Staining results of the tumours from patients with primary and recurrent cervical cancer

| Antibody/scoring | Primary disease *n* = 44 (%) | Recurrent disease *n* = 29 (%) |
|---|---|---|
| bcl-2 –ve | 10 (23) | 19 (65) |
| bcl-2 < 10% | 19 (43) | 4 (14) |
| bcl-2 11–30% | 7 (16) | 2 (7) |
| bcl-2 31–50% | 2 (4) | 0 |
| bcl-2 > 51% | 6 (14) | 4 (14) |
| mcl-1 –ve | 1 (2) | 0 |
| mcl-1 < 10% | 0 | 0 |
| mcl-1 11–30% | 1 (2) | 1 (4) |
| mcl-1 31–50% | 5 (11) | 1 (4) |
| mcl-1 51–70% | 12 (27) | 5 (17) |
| mcl-1 > 71% | 25 (57) | 21 (75) |
| bax –ve | 2 (4) | 0 |
| bax < 10% | 11 (24) | 3 (10) |
| bax 11–30% | 12 (26) | 7 (24) |
| bax 31–50% | 4 (9) | 5 (17) |
| bax 51–70% | 8 (17) | 5 (17) |
| bax > 71% | 9 (20) | 9 (31) |
| p53 –ve | 28 (64) | 15 (52) |
| p53 < 10 nuclei | 1 (2) | 5 (17) |
| p53 10 + nuclei (< 5%) | 8 (18) | 6 (21) |
| p53 > 5% | 7 (16) | 3 (10) |

**Table 3** Fisher's exact probability of an association between any two markers

| Primary/recurrent | bcl-2 | mcl-1 | bax | p53 |
|---|---|---|---|---|
| **bcl-2** | | 0.26 | 0.15 | 0.66 |
| **mcl-1** | 0.28 | | 0.62 | 0.6 |
| **bax** | 0.23 | 0.26 | | 0.45 |
| **p53** | 0.32 | 0.25 | 0.38 | |

The results for primary disease are shown above and to the right of the diagonal, whereas results for recurrent disease are shown below and to the left of the diagonal.

for the research relating to the immunohistochemical analysis of the archival material and its correlation with survival data.

Fifty-seven women with primary disease and 42 women with recurrent disease were recorded. The median age of the primary group was 43 years and the median age of the recurrent group was 45 years. The median follow-up of women with primary disease was 35 months (range 1–74 months). The 5-year survival of the primary cancer group was 59% with the median follow-up not yet reached. The median survival of women with recurrent cervical cancer was 22 months from initial diagnosis (range 2–149 months). In the primary group, there were 28 women with FIGO stage I disease, 11 with stage II disease, ten with stage III disease, five with stage IV disease and no stage was recorded in three cases. The pathology in 36 women was squamous-cell carcinoma, adenocarcinoma in eight cases, adenosquamous carcinoma in nine cases and not recorded in four cases. In the recurrent group, 19 patients initially had a stage I lesion, 13 had stage II disease, five had stage III disease, four had stage IV disease and no stage was recorded in one case. The pathology in 30 women was squamous-cell carcinoma, adenocarcinoma in six cases, adenosquamous carcinoma in five cases and not recorded in one case.

Sections (3 μm) of cervical cancer were dewaxed and endogenous peroxidase activity was blocked with 1.5% hydrogen peroxide in methanol.

The pressure cooker antigen retrieval system was used for the bcl-2 and mcl-1 antibodies (Norton et al, 1994). The primary antibody was added at the appropriate dilution (Table 1) and then incubated. The immunohistochemical procedure in this study used the *a*vidin–*b*iotin *c*omplex method (Hsu et al, 1981) using Vectastain® Universal Elite ABC kit (Vector Laboratories, Burlingame, CA, USA). The appropriately diluted primary antibody was labelled with a biotinylated secondary antibody. This was then coupled with a preformed avidin horseradish peroxidase complex and visualized with chromogen diaminobenzidine tetrahydrochloride. Appropriate positive controls were used as detailed in Table 1. The primary antibody was omitted for the negative control.

All slides were evaluated for immunostaining without knowledge of the clinical outcome. Sections were scored by determining the proportion of stained cells relative to the overall number of tumour cells, excluding those in areas of necrosis. p53 was scored in four categories: negative; fewer than ten nuclei positive; more than ten nuclei positive but less than 5% of the whole tumour positive; and more than 5% of the whole tumour positive. This type of scoring included two different ways of expressing positivity, i.e. absolute numbers of positive cells and percentage positivity, reflecting the scoring systems in published work. bcl-2 was scored in five categories: negative; less than 10% of the tumour; 10–30%; 30–50%; and > 50%. bax and mcl-1 had the same scoring system as bcl-2 with an additional positive category of >70% staining. The sections were scored independently (RAFC and CC). Disagreement over score was found with less than 10% of the slides examined and consensus was reached on further review. Positive staining was scored when the colour intensity was equivalent to the control. No attempt was made to grade intensity in this study.

### Statistical analysis

The percentage survival for each group was calculated using the Kaplan–Meier method and comparisons between groups were performed using the log-rank test (SPSS for Windows, 6.1.3 1995). When data did not extend to 5 years, survival rate is given per years of follow-up in that group.

© Cancer Research Campaign 1998

**Figure 1** bcl-2 staining and survival in primary cervical cancer. Kaplan–Meier survival curves for patients with primary cervical cancer stained with bcl-2 at a threshold of 10%. bcl-2 negative, *n* = 29, 13 events, 4-year survival 53%; bcl-2 positive, *n* = 15, two events, 4-year survival 84%, *P* = 0.03. - - -, bcl-2 stain > 10%, *n* = 15; —, bcl-2 stain < 10%, *n* = 29

**Figure 2** Graph showing survival associated with positive p53 nuclear staining using an absolute threshold (> 10 nuclei positive). p53-negative tumours, *n* = 29, six events, 4-year survival 78%; p53-positive tumours, *n* = 15, nine events, 4-year survival 38%, *P* = 0.02. - - -, p53 stain positive, *n* = 15; —, p53 stain < 10 nuclei positive, *n* = 29

### RESULTS

Suitable tissue for immunohistochemical analysis was available in 44 cases of primary cancer and 29 cases of recurrent cancer. Details of the extent of staining for the antibodies are shown in Table 2. bcl-2 positivity (> 10% of the tumour) was recorded in 34% (15/44) of primary and 21% (6/29) of recurrent tumours. mcl-1 positivity (> 70% of the tumour) was recorded in 57% (25/44) of primary and 75% (21/28) of recurrent cancers. bax staining (> 50% of the tumour) was seen in 37% (17/46) of primary and 48% (14/29) of recurrent cancers. Using an absolute method of scoring (more than ten nuclei positive), p53 was positive in 34% (15/44) of primary and 31% (9/29) of recurrent tumours. Using the semiquantitative measure of 5%, only 16% (7/44) of primary and 30% (3/10) of recurrent cancers were positive for p53.

The relationships between markers are shown in Table 3. There was no significant association among bcl-2 and the other markers.

Analysis of the immunohistochemical markers with respect to survival is shown in Table 4. bcl-2 positivity was prognostic of a good outcome in primary disease (Figure 1), whereas this was not significant in recurrent disease (*P* = 0.47). Neither mcl-1 nor bax staining was prognostic of a survival advantage. Using the threshold of ten nuclei positive, p53 was a significant prognostic

**Table 4** Survival statistics for immunohistochemical markers in primary and recurrent cervical cancers

| Antibody | Primary disease: Number of cases; events; log-rank $\chi^2_{d.f.=1}$; survival | Primary disease: *P*-value | Recurrent disease: Number of cases; events; log-rank $\chi^2_{d.f.=1}$ survival | Recurrent disease: *P*-value |
|---|---|---|---|---|
| bcl-2 | 4.46 | 0.03 | 0.52 | 0.47 |
| Staining < 10% | *n* = 29; 13 events; 4 years, 53% | | *n* = 23; 19 events; median 8 months | |
| Staining > 10% | *n* = 15; two events; 4 years, 84% | | *n* = 4; four events; median 2 months | |
| mcl-1 | 0.08 | 0.78 | 0.18 | 0.67 |
| Staining < 70% | *n* = 19; six events; 5 years, 61% | | *n* = 7; six events; median 8 months | |
| Staining > 70% | *n* = 25; nine events; 5 years, 63% | | *n* = 21; 19 events; median 8 months | |
| bax | 0.51 | 0.47 | 0.47 (at staining threshold of 50%) | 0.49 |
| Staining < 70% | *n* = 35; 11 events; 5 years, 66% | | *n* = 15; 12 events; median 8 months | |
| Staining > 70% | *n* = 9; four events; 5 years, 53% | | *n* = 14; 13 events; median 8 months | |
| p53 | 5.84 | 0.02 | 2.36 | 0.12 |
| < 10 nuclei positive | *n* = 29; six events; 4 years, 78% | | *n* = 20; 16 events; median 8 months | |
| > 10 nuclei positive | *n* = 15; nine events; 4 years, 38% | | *n* = 9; nine events; median 3 months | |

© Cancer Research Campaign 1998

marker for poor outcome in primary disease (Figure 2). p53 showed a trend towards being a significant prognostic factor in recurrent disease using this threshold. When using the semiquantitative 5% threshold, there was no significant prognostic effect with either the primary or the recurrent groups.

### DISCUSSION

In this study, bcl-2-positive staining in the cervical carcinoma was a significant prognostic factor in predicting survival in primary cervical cancer: there was an 84% 4-year survival in bcl-2-positive patients compared with a 53% 4-year survival in bcl-2-negative patients ($P = 0.03$). This finding agrees with Tjalma et al (1997) who found a survival advantage in patients with bcl-2 staining of cervical carcinoma in surgically treated disease. Tjalma et al (1998) found on multivariate analysis that survival could be independently predicted by bcl-2 expression, FIGO stage and lymphatic permeation of the tumour. Uehara et al (1995) found no survival advantage with bcl-2 staining or any evidence of interaction with bax in early-stage cervical cancer. However, in Uehara's paper it was not clear what constituted a positive result; this factor has a substantial bearing on the prognostic value of immunohistochemistry.

The lack of prognostic value of mcl-1 and bax is not surprising when one considers the widespread nature of the staining seen (mcl-1 > 10%, 43/44; bax > 10%, 33/46). This reflects the biology of the apoptotic proteins that are present in the cytoplasm even when the cell is not undergoing apoptosis (Vaux and Strasser, 1996). These proteases can be activated without having to be newly synthesized, and apoptosis can be induced without influencing transcription from the protease genes. Also, individual bcl-2 family members may have different apoptosis-triggering stimuli, activating distinct cysteine proteases. There may be partial redundancy between the activities of different proteases. Immunohistochemistry can detect proteins that are demonstrably present but may be inactive in carcinogenesis.

p53 staining was a significant prognostic factor when using an absolute method of scoring ($P = 0.02$), although Wynford-Thomas (1992) pointed out that false-positive immunostaining may occur. In addition, the antibody may detect the higher concentration of normally active wild-type p53 needed for DNA repair in that particular cell. It is, therefore, more appropriate to use the semiquantitative measure (at 5%), and to disregard the significant results relating to survival for p53 as measured by a small number of nuclei stained (< 5%). When a semiquantitative measure (> 5%) was applied, the prognostic effect was not significant ($P = 0.64$), which is similar to other reports (Hunt et al, 1996).

van Diest et al (1997) highlight the problems with scoring immunohistochemistry and suggest a protocol to reduce the variability between published papers. In this study, we have used only the defined staining pattern for each antibody, reviewed the complete tumour on the section stained and used a cut-off determined by clinical parameters. van Diest et al (1997) suggest that, in addition, the area of the lesion to be assessed and method of sampling of fields of vision should be defined. Further influences on the results of immunohistochemistry relating to tissue collection and processing can only be reduced when agreement relating to protocols is reached.

In recurrent disease, there was no prognostic significance associated with any of the markers. The recurrent group was very heterogeneous, including patients with persistent disease following primary treatment, true recurrence following a period of remission and even patients who received inappropriate primary treatment. This heterogeneity might explain why the apoptotic proteins were of no value in predicting outcome.

HPV in cervical carcinoma has been extensively investigated and is pivotal in the development of the cancer. In this study, in excess of 90% of the tumours were virus positive (unpublished results) similar to previous reports (Walboomers and Meijer, 1997). It is likely that HPV acts at an earlier point in carcinogenesis than the apoptotic proteins and, therefore, this immunohistochemical study will not detect an interaction.

Saegusa et al (1995) examined the relationship between bcl-2, bax and HPV expression. bcl-2 expression was greatest in CIN 3 compared with invasive carcinoma. However, as in the present study, this was a cross-sectional study and it is not clear whether those bcl-2-positive preinvasive cases would have progressed to cancer or not without treatment. Twenty per cent of the carcinomas in this study stained for bcl-2 and 40% for bax, similar to the present study. There was no association among bcl-2, bax staining and HPV status in the 60 squamous cancers examined.

There appeared to be no value in considering combinations of staining characteristics. The relative ratios of the proteins are important (Kernohan and Cox, 1996) and the methodology to determine this needs to be further refined. However, on the basis of this study, there does not appear to be any value in analysing the tumours with the bank of immunohistochemical markers we proposed.

In conclusion, the identification of bcl-2 expression as determined by immunohistochemistry is a useful prognostic factor in cervical cancer. Although bcl-2 prevents apoptosis, it is not clear why this should then lead to a better outcome. The addition of further immunohistochemical markers related to apoptosis does not appear to be helpful, although the biology of the bcl-2 family suggests that the interaction of pro- and anti-apoptotic factors should be important.

### ACKNOWLEDGEMENTS

The work was supported by a grant from the Cancer Research Committee of St. Bartholomew's Hospital, London.

### REFERENCES

van Diest PJ, van Dam P, Henzen-Logmans SC, Berns E, van der Burg MEL, Green J and Vergote I (1997) A scoring system for immunohistochemical staining: consensus report of the task force for basic research of the EORTIC-GCCG. *J Clin Pathol* **50**: 801–804

Haldar S, Negrini M, Monne M, Sabbioni S and Croce CM (1994) Down-regulation of bcl-2 by p53 in breast cancer cells. *Cancer Res* **54**: 2095–2097

Hall PA, Meek D and Lane DP (1996) p53-integrating the complexity. *J Pathol* **180**: 1–5

Henriksen R, Wilander E and Öberg (1995) Expression and prognostic significance of Bcl-2 in ovarian tumours. *Br J Cancer* **72**: 1324–1329

Hockenbery DM, Nuñez G, Milliman C, Schreiber RD and Korsmeyer SJ (1990) Bcl-2 is an inner mitochondrial membrane protein that blocks programmed cell death. *Nature* **348**: 334–336

Hsu S-M, Raine L and Fanger H (1981) A comparative study of the peroxidase–antiperoxidase method and an avidin–biotin complex method for studying polypeptide hormones with radioimmunoassay antibodies. *Am J Clin Pathol* **75**: 734–738

Hunt CR, Hale RJ, Buckley CH and Hunt J (1996) p53 expression in carcinoma of the cervix. *J Clin Pathol* **49**: 971–974

Kernohan NM and Cox LS (1996) Regulation of apoptosis by bcl-2 and its related proteins: immunochemical challenges and their therapeutic implications. *J Pathol* **179**: 1–3

© Cancer Research Campaign 1998 

Krajewski S, Bodrug S, Gascoyne R, Berean K, Krajewski M and Reed JC (1994) Immunohistochemical analysis of Mcl-1 and Bcl-2 proteins in normal and neoplastic lymph nodes. *Am J Pathol* **145**: 515–525

Krajewski S, Bodrug S, Krajewski M, Shabaik A, Gascoyne R, Berean K and Reed JC (1995) Immunohistochemical analysis of Mcl-1 protein in human tissues. Differential regulation of mcl-1 and bcl-2 protein production suggests a unique role for mcl-1 in control of programmed cell death in vivo. *Am J Pathol* **146**: 1309–1319

Miyashita T, Krajewski S, Krajewski M, Wang H, Lin HK, Hoffman B, Lieberman D and Reed JC (1994) Tumour suppressor *p53* is a regulator of *bcl*-2 and *bax* gene expression *in vitro* and *in vivo*. *Oncogene* **9**: 1799–1805

Nguyen PL, Zukerberg LR, Benedict WF and Harris NL (1996) Immunohistochemical detection of p53, bcl-2, and retinoblastoma proteins in follicular lymphoma. *Am J Clin Pathol* **105**: 538–543

Norton AJ, Jordan S and Yeomans P (1994) Brief, high-temperature heat denaturation (pressure cooking): a simple and effective method of antigen retrieval for routinely processed tissues. *J Pathol* **173**: 371–379

Oltvai ZN and Korsmeyer SJ (1994) Checkpoints of duelling dimers foil death wishes. *Cell* **79**: 189–192

Saegusa M, Takano Y, Hashimura M, Shoji Y and Okayasu I (1995) The possible role of bcl-2 expression in the progression of tumors of the uterine cervix. *Cancer* **76**: 2297–2303

Tjalma W, De Cuyper E, Weyler J, Van Marck E, De Pooter C, Albertyn G and van Dam P (1998) Expression of bcl-2 in invasive and in situ carcinoma of the uterine cervix. *Am J Obstet Gynecol* **178**: 113–117

Tjalma W, Weyler J, Goovaerts G, De Pooter C, Van Marck E and van Dam P (1997) Prognostic value of bcl-2 expression in patients with operable carcinoma of the uterine cervix. *J Clin Pathol* **50**: 33–36

Uehara T, Kuwashima, Izumo T, Kishi K, Shiromizu K and Matsuzawa M (1995) Expression of the proto-oncogene *bcl-2* in uterine cervical squamous cell carcinoma: its relationship to clinical outcome. *Eur J Gynaecol Oncol* **16**: 453–460

Vaux DL and Strasser A (1996) The molecular biology of apoptosis. *Proc Natl Acad Sci USA* **93**: 2239–2244

Walboomers JMM and Meijer CJLM (1997) Do HPV-negative cervical carcinomas exist? *J Pathol* **181**: 253–254

Wynford-Thomas D (1992) p53 in tumour pathology: can we trust immunocytochemistry? *J Pathol* **166**: 329–330

Yang E and Korsmeyer SJ (1996) Molecular thanatopsis: a discourse on the bcl-2 family and cell death. *Blood* **88**: 386–401

Yang E, Zha J, Jockel, J, Boise LH, Thompson CB and Korsmeyer SJ (1995) Bad, a heterodimeric partner for bcl-$X_L$ and bcl-2, displaces bax and promotes cell death. *Cell* **80**: 285–291

© Cancer Research Campaign 1998


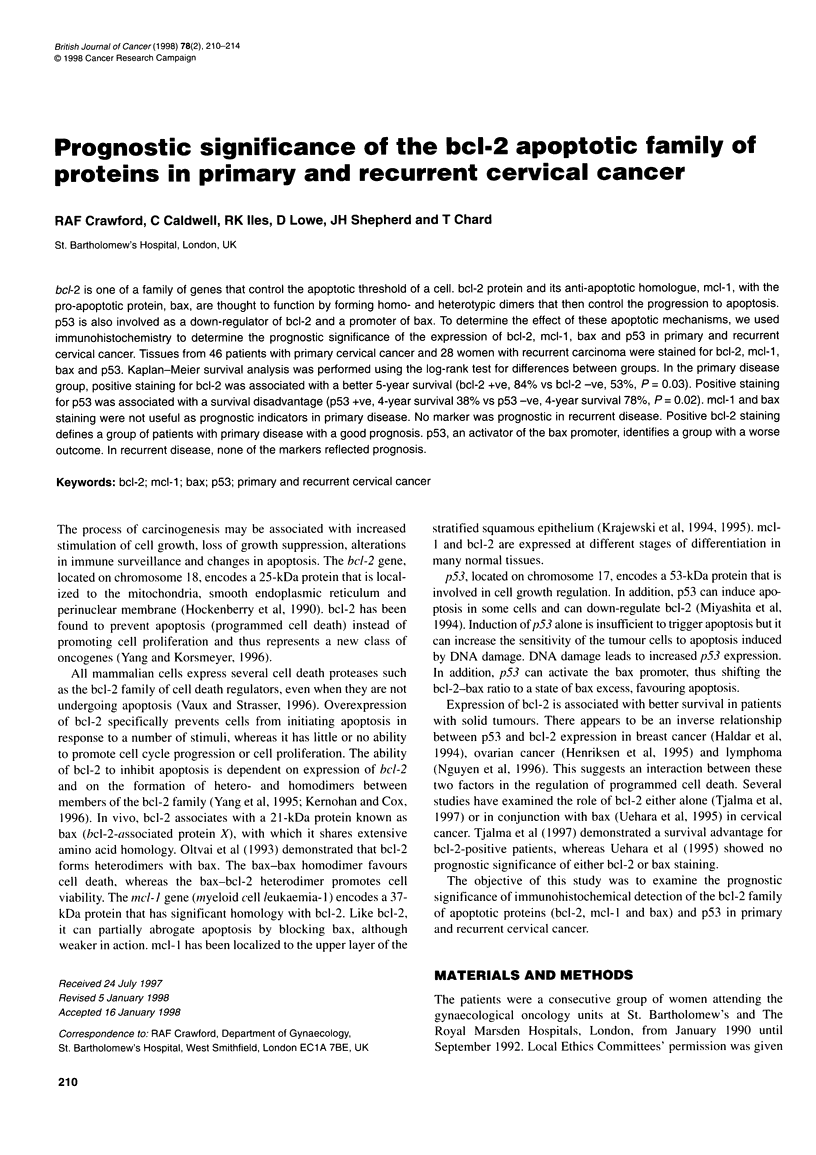

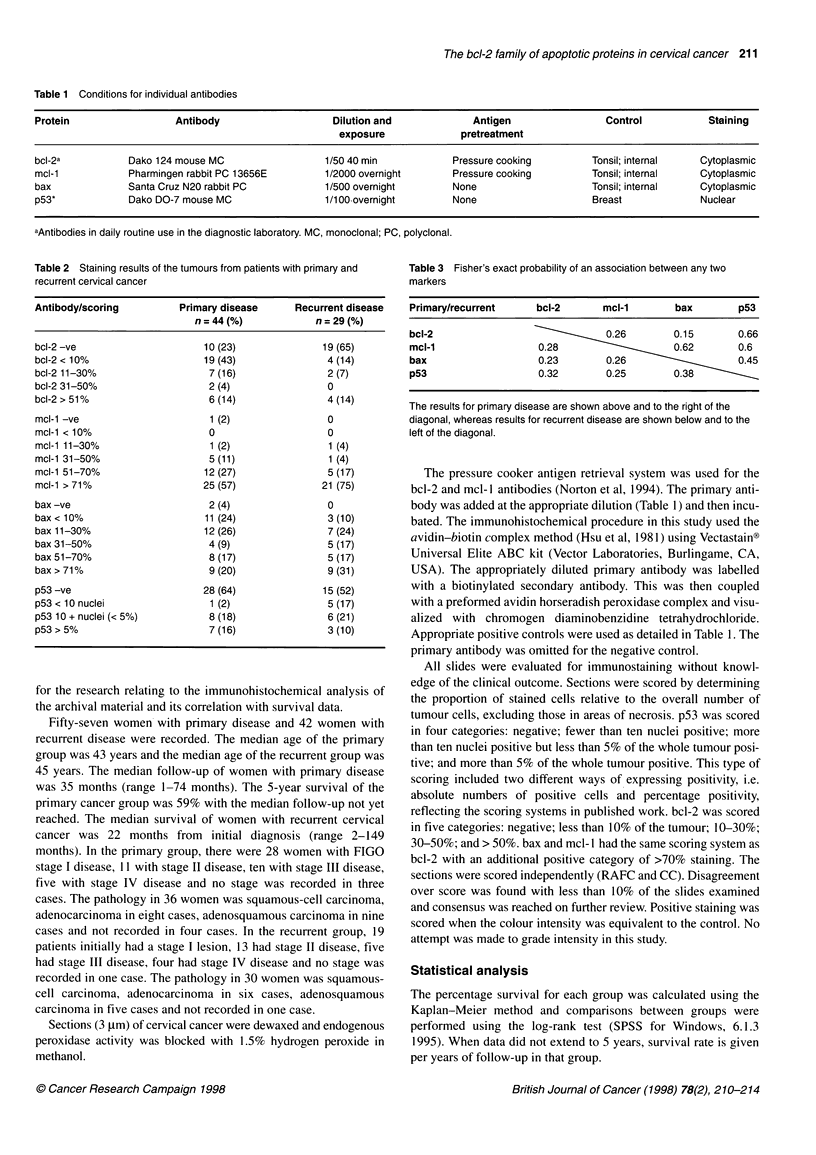

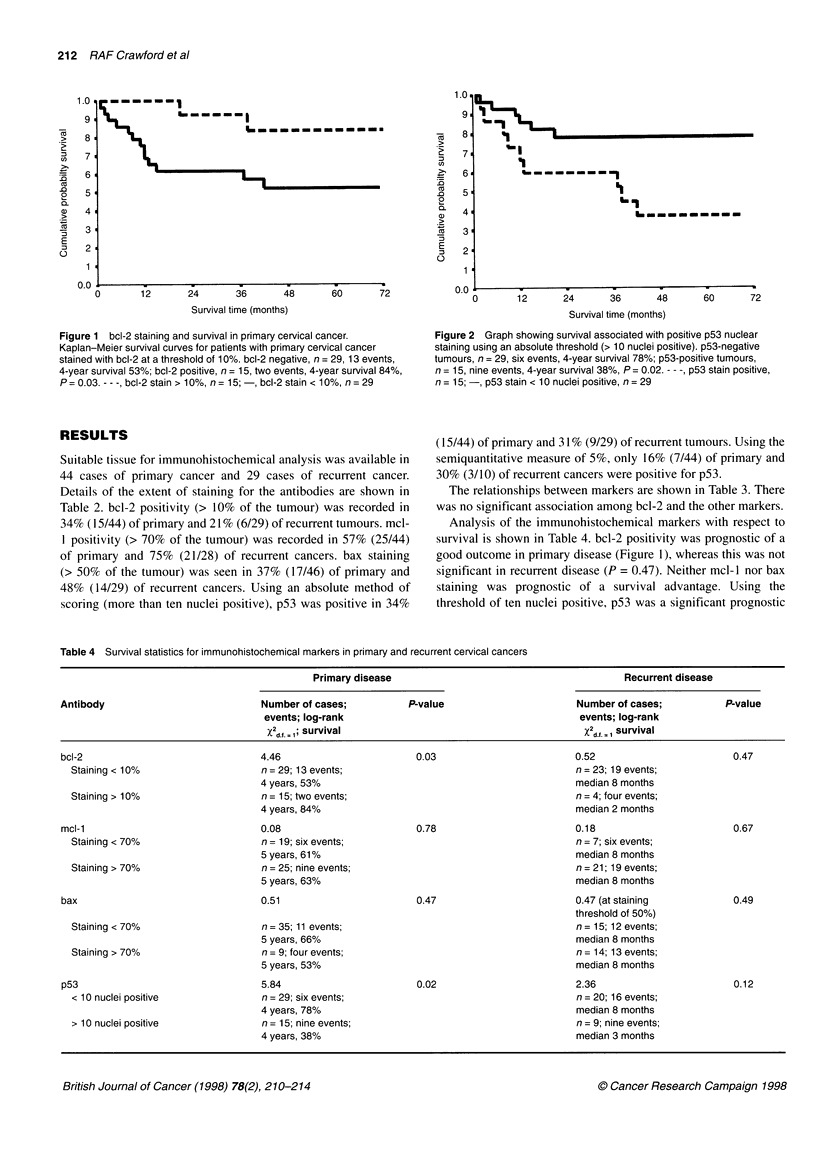

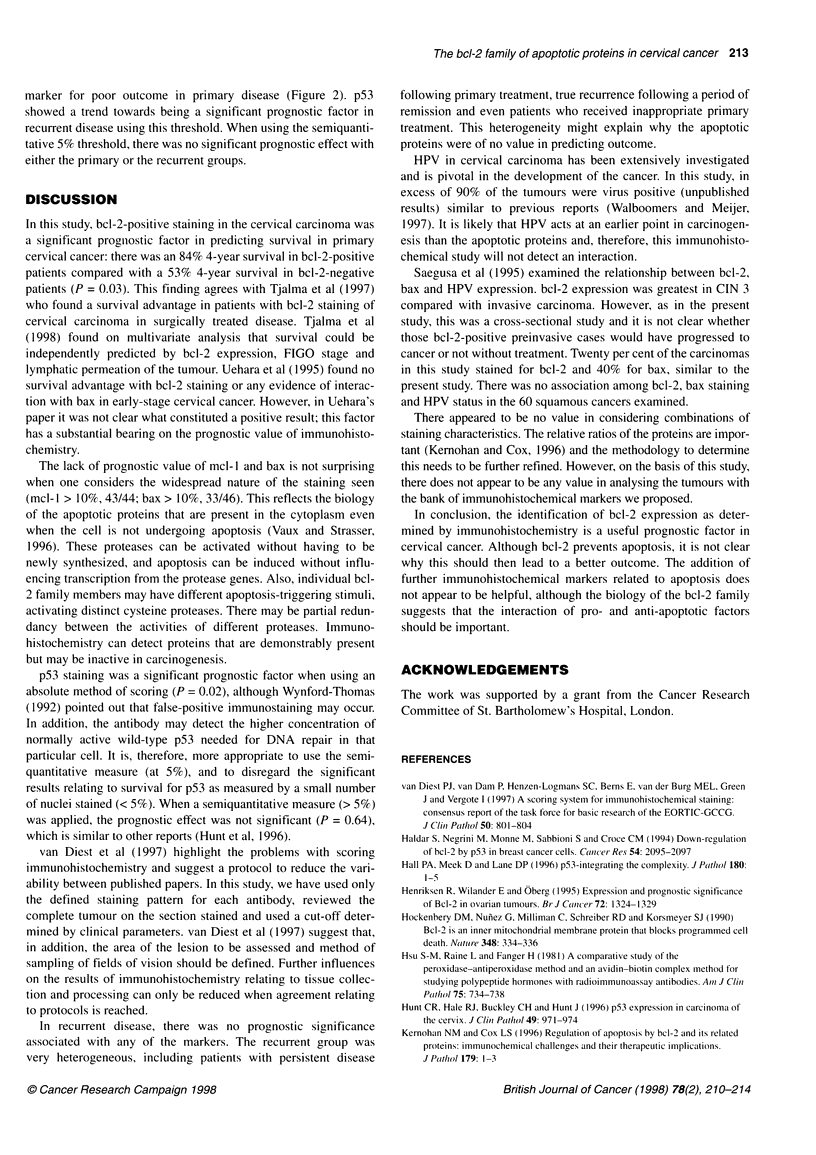

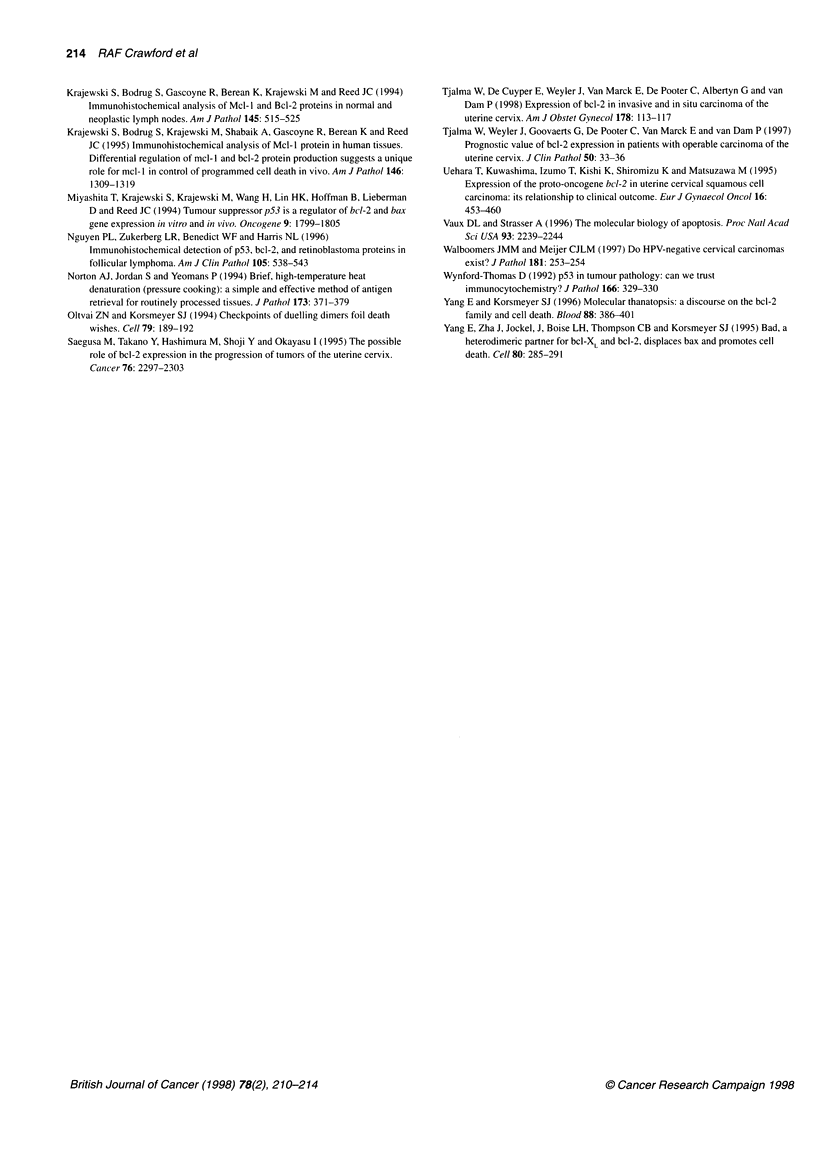


## References

[OCR_00516] Haldar S., Negrini M., Monne M., Sabbioni S., Croce C. M. (1994). Down-regulation of bcl-2 by p53 in breast cancer cells.. Cancer Res.

[OCR_00524] Henriksen R., Wilander E., Oberg K. (1995). Expression and prognostic significance of Bcl-2 in ovarian tumours.. Br J Cancer.

[OCR_00528] Hockenbery D., Nuñez G., Milliman C., Schreiber R. D., Korsmeyer S. J. (1990). Bcl-2 is an inner mitochondrial membrane protein that blocks programmed cell death.. Nature.

[OCR_00533] Hsu S. M., Raine L., Fanger H. (1981). A comparative study of the peroxidase-antiperoxidase method and an avidin-biotin complex method for studying polypeptide hormones with radioimmunoassay antibodies.. Am J Clin Pathol.

[OCR_00540] Hunt C. R., Hale R. J., Buckley C. H., Hunt J. (1996). p53 expression in carcinoma of the cervix.. J Clin Pathol.

[OCR_00544] Kernohan N. M., Cox L. S. (1996). Regulation of apoptosis by Bcl-2 and its related proteins: immunochemical challenges and therapeutic implications.. J Pathol.

[OCR_00553] Krajewski S., Bodrug S., Gascoyne R., Berean K., Krajewska M., Reed J. C. (1994). Immunohistochemical analysis of Mcl-1 and Bcl-2 proteins in normal and neoplastic lymph nodes.. Am J Pathol.

[OCR_00558] Krajewski S., Bodrug S., Krajewska M., Shabaik A., Gascoyne R., Berean K., Reed J. C. (1995). Immunohistochemical analysis of Mcl-1 protein in human tissues. Differential regulation of Mcl-1 and Bcl-2 protein production suggests a unique role for Mcl-1 in control of programmed cell death in vivo.. Am J Pathol.

[OCR_00566] Miyashita T., Krajewski S., Krajewska M., Wang H. G., Lin H. K., Liebermann D. A., Hoffman B., Reed J. C. (1994). Tumor suppressor p53 is a regulator of bcl-2 and bax gene expression in vitro and in vivo.. Oncogene.

[OCR_00570] Nguyen P. L., Zukerberg L. R., Benedict W. F., Harris N. L. (1996). Immunohistochemical detection of p53, bcl-2, and retinoblastoma proteins in follicular lymphoma.. Am J Clin Pathol.

[OCR_00575] Norton A. J., Jordan S., Yeomans P. (1994). Brief, high-temperature heat denaturation (pressure cooking): a simple and effective method of antigen retrieval for routinely processed tissues.. J Pathol.

[OCR_00580] Oltvai Z. N., Korsmeyer S. J. (1994). Checkpoints of dueling dimers foil death wishes.. Cell.

[OCR_00584] Saegusa M., Takano Y., Hashimura M., Shoji Y., Okayasu I. (1995). The possible role of bcl-2 expression in the progression of tumors of the uterine cervix.. Cancer.

[OCR_00589] Tjalma W., De Cuyper E., Weyler J., Van Marck E., De Pooter C., Albertyn G., van Dam P. (1998). Expression of bcl-2 in invasive and in situ carcinoma of the uterine cervix.. Am J Obstet Gynecol.

[OCR_00594] Tjalma W., Weyler J., Goovaerts G., De Pooter C., Van Marck E., van Dam P. (1997). Prognostic value of bcl-2 expression in patients with operable carcinoma of the uterine cervix.. J Clin Pathol.

[OCR_00599] Uehara T., Kuwashima Y., Izumo T., Kishi K., Shiromizu K., Matsuzawa M. (1995). Expression of the proto-oncogene bcl-2 in uterine cervical squamous cell carcinoma: its relationship to clinical outcome.. Eur J Gynaecol Oncol.

[OCR_00605] Vaux D. L., Strasser A. (1996). The molecular biology of apoptosis.. Proc Natl Acad Sci U S A.

[OCR_00609] Walboomers J. M., Meijer C. J. (1997). Do HPV-negative cervical carcinomas exist?. J Pathol.

[OCR_00613] Wynford-Thomas D. (1992). P53 in tumour pathology: can we trust immunocytochemistry?. J Pathol.

[OCR_00617] Yang E., Korsmeyer S. J. (1996). Molecular thanatopsis: a discourse on the BCL2 family and cell death.. Blood.

[OCR_00621] Yang E., Zha J., Jockel J., Boise L. H., Thompson C. B., Korsmeyer S. J. (1995). Bad, a heterodimeric partner for Bcl-XL and Bcl-2, displaces Bax and promotes cell death.. Cell.

[OCR_00510] van Diest P. J., van Dam P., Henzen-Logmans S. C., Berns E., van der Burg M. E., Green J., Vergote I. (1997). A scoring system for immunohistochemical staining: consensus report of the task force for basic research of the EORTC-GCCG. European Organization for Research and Treatment of Cancer-Gynaecological Cancer Cooperative Group.. J Clin Pathol.

